# The Book World of Medicine and Science

**Published:** 1895-04-13

**Authors:** 


### The Book World of Medicine and Science.

Aseptic Treatment of Wounds. By Dr. Schimmelbusch. Translated by Alfred Rake, M.B., F.R.C.S. (London: H. K. Lewis. 1894. 250 pages, 40 illustrations.)

Dr. Schimmelbusch's work on the aseptic treatment of wounds is a scientific work of very great value, and teems with interesting facts. While confirming some old-established ideas, it dissipates others entirely. He gives us, too, a short account of the life history of those germs whose biology is known. In the preface, the translator, Dr. Alfred Rake, draws attention to some of the causes which operate against the efficient carrying out of aseptic methods in England. Prominent among these is the frequent change of dressers and house surgeons, who have no time to acquire the true spirit of aseptism. Turning to the main object of the book—the treatment of wounds, we see many axioms which we wish were written in the minds of all surgeons. We quote a few: "The first duty of a surgeon is to keep his wounds free from pathogenic organisms." A surgeon should just as carefully purify his hands before dressing a septic wound as an aseptic one, for "even wounds that are already diseased can be still further infected." "Practically, all infection is by contact.' The directions for the avoidance of infection of wounds are most minute, and easy to understand. It is only when he urges the use of heat as a steriliser, and boiling in soda solution, in the place of the carbolic solutions recommended by Lister, and aseptic moss dressings in the place of antiseptic dressings, that we join issue with him. To quote him against himself, he says, on page 190, "Absolute freedom from germs cannot be counted on, even with strict asepsis." Dr. Schimmelbusch is at great pains to prove that nearly all the details of Lister's system of treating wounds are open to theoretical objections. However strong these objections may be theoretically, there remains, however, the fact that there are at the present time surgeons all over Great Britain who have been trained by Lister or his assistants, who are daily operating with uniformly aseptic results, and that they can count on those results with the most absolute certainty. Again, as showing the absolute safety and ease with which Listerism (*i.e.* asepsis by means of the use of chemicals) can be carried out, the writer has seen cases admitted with erysipelas, nursed in Lister's general surgical ward. Dr. Schimmelbusch admits that this is impossible with his system of asepsis, for on page 174, after recommending a special theatre for changing dressings, he says, "It is almost more urgently necessary that there should be different and separate wards for patients with healthy wounds, and for those whose wounds are infected, than to have different theatres. It is as necessary to have special wards for septic surgical cases, as it is to have them for patients suffering from typhoid or tuberculosis of the lungs." Many of the arguments which he urges against Listerism really apply to its abuse, not its use. Nevertheless we would bring this book to the notice of surgeons generally, for the facts which it relates, and the way in which they are presented, may lead many a step further on the road to true asepsis.

FOOD FOR THE SICK AND CONVALESCENT. By MARY PEACHEM, Diplomée First-Class, South Kensington. (Printed by Messrs. Rose and Harris, Broadmead, Bristol. Price 1s.)

This is a clearly printed little pamphlet, consisting of eight pages of recipes for the preparation of nourishment for invalids. All kinds of broths, drinks, and puddings are given, as well as directions for cooking fish, game, and poultry, &c. The information is put in a concise form, which adds greatly to the practical utility of pages which will be valued by private and district nurses, and by many others, who will find numerous suggestions for variable and digestible sick-room diets.

